# A Simple Novel Technique of Infrared Meibography by Means of Spectral-Domain Optical Coherence Tomography: A Cross-Sectional Clinical Study

**DOI:** 10.1371/journal.pone.0165558

**Published:** 2016-10-31

**Authors:** Pietro Emanuele Napoli, Franco Coronella, Giovanni Maria Satta, Claudio Iovino, Raffaele Sanna, Maurizio Fossarello

**Affiliations:** Department of Surgical Sciences, Eye Clinic, University of Cagliari, Cagliari, Italy; Charite Universitatsmedizin Berlin, GERMANY

## Abstract

**Purpose:**

To compare a novel spectral-domain optical coherence tomography (SD-OCT) technique with traditional lid transillumination for evaluation of meibomian glands (MGs) and to assess the relation of MG morphologic changes to the glandular atrophy.

**Design:**

Evaluation of diagnostic technology.

**Participants:**

Sixty-one patients with obstructive MGD (30 men, 31 women; age [mean ± standard deviation] 45.1 ± 12.1 years), and 75 control subjects (32 men, 43 women; 44.1 ± 12.5 years) were recruited in order to have a balanced distribution of glandular features.

**Methods:**

Agreement between SD-OCT and lid transillumination examination for the detection of drop-out (partial or complete loss of MGs) and microscopic changes (i.e. shortening, distortion, segmentation and entanglement), as well as the relationship between morphological features and MG atrophy were evaluated.

**Main Outcome Measures:**

Agreement between the two meibographic techniques, bias in symmetry of classification, and association analysis between microscopic changes and MG dropout.

**Results:**

Overall agreement for *all* morphological features was substantial (Cohen kappa coefficient = 0.77; p<0.001), even if, the majority of disagreement occurred for cases with *segmentation*, where agreement was present in only 108 (81.82%) of 132 eyes with adequate images for interpretation, and where SD-OCT tended to diagnose more cases not detected by traditional lid transillumination (McNemar test, p<0.001). Moreover, segmentation and distortion pattern negatively correlated with the degree of drop-out, whereas shortening and entanglement pattern demonstrated only a weak correlation (Spearman’s ρ was -0.691, -0.491, -0.359, -0.385, respectively).

**Conclusions:**

Each method has its advantages but in general there was close agreement between these meibographic techniques, particularly for MG dropout, which supports the reliability of our novel, simple and patient-friendly SD-OCT approach.

## Introduction

Meibomian glands (MGs) secrete a lipid substance, i.e. the *meibum*, which retards tear evaporation and functions as lubricants for the eyelids during blinking [1,2]. In case of absence or altered integrity of meibum, the health status of the tear film and ocular surface may change, potentially causing ocular discomfort and ocular surface disease [3,4].

Obstructive meibomian gland dysfunction (MGD) is the most common cause of lipid layer deficiency and evaporative dry eye, and often results in increased tear evaporation, decreased tear stability, loss of lubrication, and damage to the ocular surface epithelium [5,6]. Molinari and Stanek reported the prevalence of MGD to be 33% in patients younger than 30 years and 71.7% in individuals 60 years or older [7].

In clinical practice, there are several methods for assessment of MG status and function, such as slit-lamp examination of the lid margins and ocular surface epithelium, meibometry, evaluation of meibum properties, and meibography [8–14]. Among them, meibography is the only approach that can show some important details of the silhouette of MGs, i.e. structural appearance and atrophy [15]. In particular, it has been shown that some morphological features of MGs, i.e. drop-out, shortening and distortion, are significantly associated with MGD [16]. For all these reasons, the practice of lid transillumination for observing MG rows remains a key technique (conventional or standard technique) in studying atrophic processes involving MGs, as well as their gross and microscopic changes [17–18].

However, although meibography was developed > 30 years ago, it is not widely used, probably because of necessary of examiner expertise as well as equipment dedicated to this analysis, which may be available only in specialized centers. Also, ocular irritation (e.g. discomfort and/or pain) induced by direct application of probe to the eyelid in transillumination systems may be a cause of the limited use of all systems of *contact meibography* [15,16].

To solve these problems, the authors introduced for the first time a simple *image processing technique* to obtain a noncontact, patient-friendly, infrared meibography by means of spectral-domain optical coherence tomography (IRM-OCT), i.e. by using the commercially available, *built-in software* to elaborate OCT images. It is interesting to note that, unlike traditional meibographic approaches, spectral-domain OCT became in the past decade one of the most widely used device in ophthalmology imaging technology for its high resolution and fast *scan-speed* (greater than time-domain OCT). Accordingly, with our IRM-OCT technique may be not necessary a meibographic system exclusively *dedicated* to MGs examination, since spectral-domain OCT can be also available to detect details of others ocular tissues in both anterior and posterior segment of eye [19–23].

The goal of this study was therefore to describe a novel technique of IRM-OCT and to determine the level of agreement between this approach and standard meibography (traditional contact method) in patients with MGD and control subjects for the detection of a number of conventional and new MGs morphological findings. We also investigated the relation of the morphological changes to the glandular atrophy, as detected by IRM-OCT.

## Methods

### Subjects and examinations

This study was approved by the Office of Research Ethics, University of Cagliari, (protocol number: 2014/2682) and it adhered to the tenets of the Declaration of Helsinki. Written informed consent was obtained from all subjects before examination.

Patients presenting with complaints of ocular irritation at the Eye Clinic, who had no history of Stevens-Johnson syndrome, Sjögren’s syndrome, systemic drugs use, contact lens wear, chemical, thermal, and radiation injury, or had not undergo any procedure or ocular surgery that would create an ocular surface problem, were considered to be potential participants of the study.

All physicians (PEN, FC, GMS) who examined study participants were specialists in the field of ocular surface. Data were obtained from the right eye of each subject. Examinations were performed sequentially as follows: clinical history, McMonnies questionnaire, slit-lamp observation of lid margin abnormalities and the ocular surface, fluorescein break-up time (FBUT), fluorescein staining of the cornea and conjunctiva graded according to the Oxford system, observation of the MGs in the upper and lower eyelids using lid transillumination (i.e. *contact meibography*) and using a novel OCT technique (i.e. *noncontact infrared meibography*), Schirmer I test, and evaluation of meibomian secretion (meibum) after digital pressure application on the upper lid tarsus [18–24]. Examinations were completed on the same day, generally within 25–30 minutes (~ 2–3 minutes for IRM-OCT, ~ 5–6 minutes for transillumination biomicroscopy). Assessment of the total area of the MGs in the everted eyelid (upper or lower) was performed, acquiring *three* scans (central, temporal and nasal) with IRM-OCT, and *six* or *seven* images (depending on the eyelid dimensions) with conventional meibography (since the transillumination area is small) [18,24].

After these clinical examinations, the candidate subjects were classified into 2 groups. The control group included subjects who fulfilled the following criteria: (1) McMonnies score < 10, (2) no tear film abnormalities (FBUT ≥ 5 seconds and Schirmer I test value ≥ 5 mm), and (3) no abnormalities of the lid margin or meibum [19,20].

The MGD group included subjects who fulfilled the diagnostic criteria for obstructive MGD: (1) chronic ocular discomfort (McMonnies questionnaire score > 10, including the positive score obtained by questions about symptoms n.5 and n.6), (2) at least one anatomic abnormalities around the meibomian gland orifices (irregularity of the lid margin, vascular engorgement, displacement of the mucocutaneous junction), (3) decreased meibum expression and obstructive findings of gland orifices [25].

Individuals whose eyes had excessive meibomian lipid secretion (seborrheic MGD), congenital deficiency of MGs, ocular allergies or any ocular disease potentially affecting tear film production or function, were excluded.

Our study groups comprised 61 patients with obstructive MGD (45.1 ± 12.1 years [mean ± SD], 50.8% female), and 75 controls (44.1 ± 12.5 years, 57.3% female). Subjects selection and clinical/instrumental exams were performed from January 2015 to September 2015.

### FBUT and fluorescein ocular surface staining

The standard FBUT measurement was performed after instillation of a 5 μl sample of 2% fluorescein preservative-free solution in the conjunctival sac with a micropipette. After instillation, a yellow filter (Kodak Wratten no. 12) was used to enhance contrast when assessing FBUT (within 10–30 sec) and staining of the ocular surface (within 1–2 min) with a biomicroscope (×10 objective, under blue-light illumination) [5]. The patients were instructed to blink several times for a few seconds to ensure adequate mixing of the dye. The interval between the last complete blink and appearance of the first dry spot at the center of the cornea was timed using a chronometer. Three evaluations of FBUT were conducted, and the mean value was taken for data analysis [26]. The extent of the corneal surface area stained was graded according to the Oxford system [5].

### Meibography by lid transillumination (standard meibography)

Meibography was performed, similarly to others, by using a transillumination device for vitrectomy with a fiberoptic light source (Millenium system, Bausch & Lomb^®^, San Leandro, CA, USA) with a 20-gauge disposable fiber light guide [24].

### Novel technique of noncontact infrared meibography by means of spectral-domain OCT

**Fig 1:**
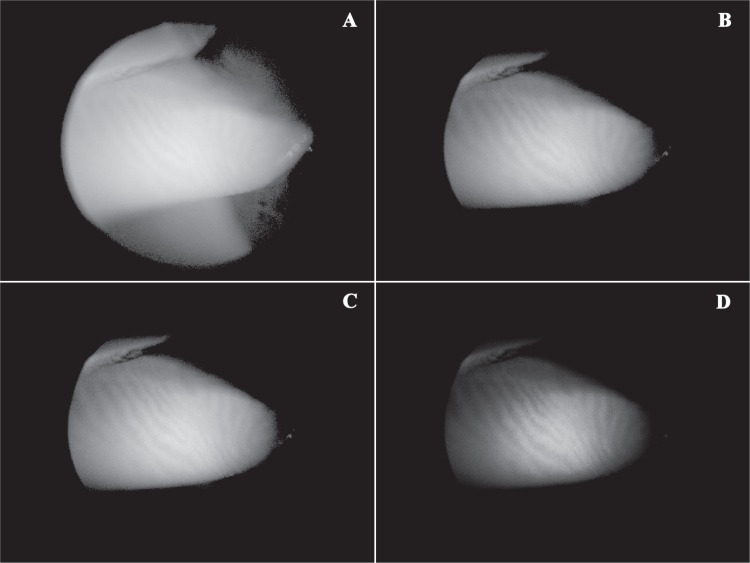
Image processing protocol for the detection of the meibomian glands by optical coherence tomography. In the raw, *unmodified* infrared image on optical coherence tomography (OCT) of the everted upper lid, i.e. without contrast/brightness adjustments, the detection of meibomian glands is difficult or nearly uninterpretable (at this time, their rows are only superficially visible). Applying our very simple *image processing protocol*, infrared imaging by OCT can easily reveal the silhouette of gland structure. Specifically, after hiding navigator (A), the image of the meibomian glands has been improved by modulating the contrast (mean percentage increase ± SD, 140.6% ± 17.7%, B) and brightness (mean percentage decrease ± SD, 6% ± 4.9%, C; see also Table 1). Selecting COLOR mode, it is also possible to obtain a false-color infrared OCT image of high quality for interpretation.

All images of MGs were acquired by using a commercially available OCT device, Cirrus^™^ HD-OCT 4000 (Carl Zeiss Meditec Inc., Dublin, California, USA), without causing patient discomfort. This imaging system is a spectral-domain OCT platform that works at a wavelength of 840 nm, takes 27,000 axial scans per second, and has a 5-μm axial resolution. The upper and lower eyelids were gently everted, taking care to expose palpebral conjunctiva as perpendicular as possible to the observer’s line of sight (when looking straight ahead), and meibomian glands imaged, despite being only superficially visible (Fig 1). The Anterior Segment 5 Line Raster scanning protocol was used to generate all infrared images. Scans were repeated until an image of satisfying quality was obtained.

Using the *built-in software* for enhancing or reducing the contrast and/or brightness of scanned images, a single analyzer (PEN) applied the *image processing protocol* as described step-by-step in details in Table 1 and Fig 1. Each scan was interpreted by a second observer, who was masked to the study, that assessed the quality of the IRM-OCT images as either adequate or *unreadable* (i.e. uninterpretable) and recorded the conjunctival reflection as mild, fair, or excessive.

**Table 1:** Reference values of the *image processing protocol* for enhancing meibomian gland observation on optical coherence tomography.

		Mean	SD	Range	Minimum	Maximum
Upper lid	*Brightness %*	-6.0	4.9	16.0	-12.5	3.5
	*Contrast %*	140.6	17.7	52.0	108.0	160.0
Lower lid	*Brightness %*	-3.01	3.05	5.8	-5.8	0.0
	*Contrast %*	87.0	24.5	57.0	59.0	116.0

% = respect to baseline/default values.

SD = standard deviation.

### Meibographic image analysis

**Fig 2:**
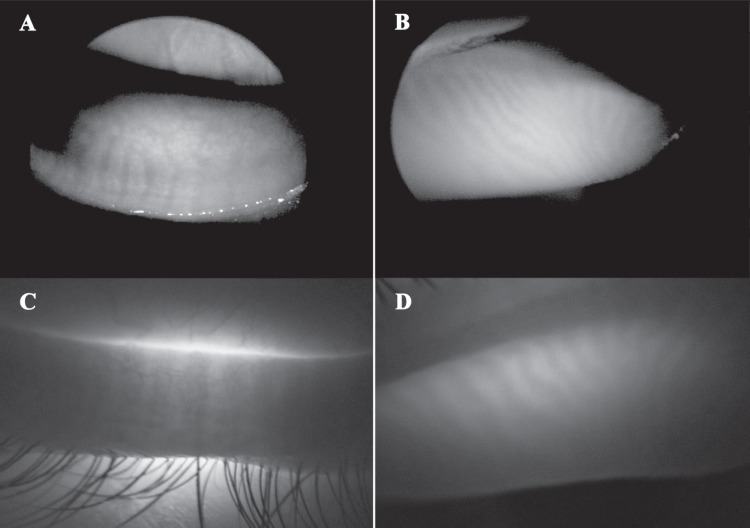
Meibomian glands of the lower and upper lid viewed by non-contact infrared meibography on optical coherence tomography (IRM-OCT) and transillumination meibography. The glandular architecture, i.e. acini and tubules, is revealed as a hyper-reflective structure (patches or rows) surrounded by hypo-reflective tissue by means of IRM-OCT (A and B, respectively, lower and upper eyelid), and, conversely, as a dark structure surrounded by grey-white tissue in transillumination (C and D, respectively, lower and upper eyelid).

Each participant was *independently* examined by 1 of 2 ocular surface experts (F.C. or G.M.S.), who performed both contact and noncontact meibographic techniques (Fig 2). Immediately after meibographic examination, the chart note from that day was reviewed by the study coordinator (P.E.N.).

### Dropout or atrophy of MGs (meiboscore)

Partial or complete loss of meibomian glands was scored, in both techniques, using the following grades for each eyelid: grade 0, no loss of meibomian glands; grade 1, area loss was less than 33% of the total meibomian gland area; grade 2, area loss was between 33% and 66%; grade 3, area loss was more than 66% [17]. The meiboscores for the upper and lower eyelids were summed to obtain a score of 0 through 6 for each eye [16].

### Microscopic morphological features of MGs

**Fig 3:**
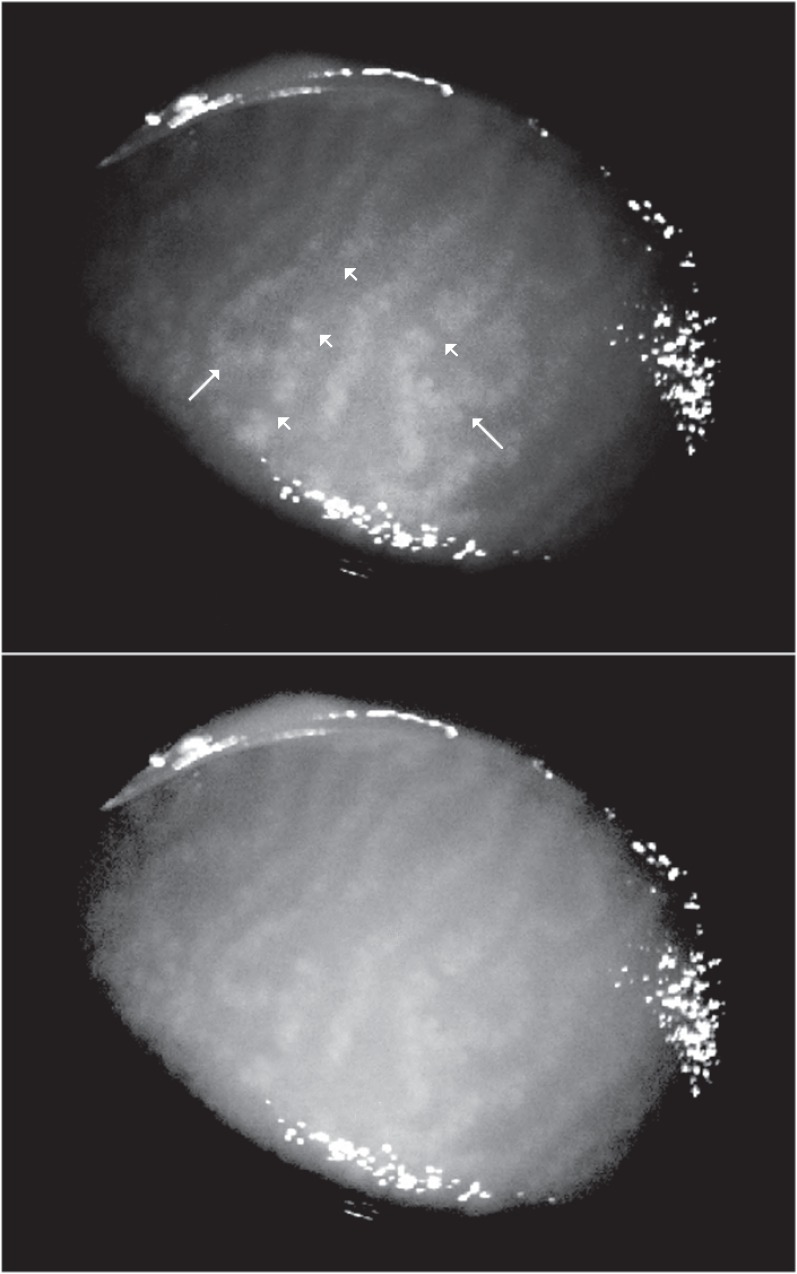
Non-contact infrared meibography obtained by optical coherence tomography (IRM-OCT) showing segmentation and entanglement of meibomian glands. Morphological analysis of glandular tissue may reveal micro-structural abnormalities as *segmentation*, i.e., the meibomian gland rows are divided into pieces along their structure (short arrows), and/or *entanglement*, i.e., the meibomian gland rows show a tangled pattern or/and hairpin-loop like changes in their structure (long arrows). False-color (top) and black/white (bottom) IRM-OCT images of the same patient (34 years old, male) with meibomian gland dysfunction.

As previously reported, shortening and distortion were evaluated as present or not present [16]. Moreover, we also introduced two new *well-defined* parameters of MG morphology, according to the following criteria: segmentation (meibomian glands appear divided into pieces in their row, Fig 3), and entanglement (meibomian glands show a tangled pattern or/and hairpin-loop like changes in their row, Fig 3), which were also evaluated as present or not present.

### Schirmer Test

For further evaluation of tears, the standard Schirmer I test without topical anesthesia was performed. The paper test strips (Alcon Laboratories, Fort Worth, TX) were placed over the lid margin at the junction of the lateral and middle thirds of the lower eyelid for 5 minutes. The millimeters of strip wetting were measured and recorded.

### Experimental procedure

All examinations were conducted in the same conditions of temperature (21.23 ± 0.47°C), humidity (40 ± 5.72%) and time of the day (between 3 PM to 5 PM) in a dimly lit consulting room.

### Statistical analysis

Descriptive statistics such as frequency, range, mean, and standard deviation were calculated for each characteristics.

The agreement of standard meibography versus infrared OCT meibography for each of the morphological features was assessed as the percentage agreement, and by calculating the Cohen kappa coefficient (unweighted and weighted). We also evaluated the ‘overall’ agreement in reporting the presence or absence of MG abnormalities, i.e. at least one of plurality of possible morphological changes (drop-out included or not). Guidelines used for interpreting the amount of agreement were those reported by Landis and Koch, where k values were defined as follows: k = 0 is poor agreement; k = 0.01–0.2 is slight agreement; k = 0.21–0.40 is fair agreement; k = 0.41–0.60 is moderate agreement; k = 0.61–0.80 is substantial agreement; and k = 0.81–1.00 is almost perfect agreement [27]. In addition, a binomial test, also known as the McNemar test, was performed as a test for bias in symmetry of the disagreements in reading of the paired images (for each parameter), and Bonferroni correction for the *post-hoc* analysis.

Relationship between presence/degree of drop-out and microscopic morphological features of MGs, as detected by IRM-OCT, was determined by calculating Pearson χ square test and Spearman’s ρ test.

With an accepted alpha risk of 0.05 and a β risk of 0.20 (i.e., 80% statistical power) in a two-sided test, approximately 60 subjects are needed before a correlation coefficient of 0.35 is said to be statistically significant.

If an infrared OCT meibography image was graded as *unreadabl****e***, then the subject’s data point for both techniques was excluded from analysis of the total agreement. Statistical significance was considered if *p* < 0.05.

## Results

### Patient data

The demographic and clinical characteristics are summarized in Table 2 and in S1 and S2 Tables. The noncontact infrared meibographic images obtained with OCT were of sufficient quality for interpretation in 132 (97.1%) of 136 cases tested. In cases of uninterpretable images, the OCT operator attributed poor image quality to artifact due to the excessive *conjunctival reflection* (Table 3).

**Table 2:** Distributions of meibomian gland abnormalities as detected by meibographic techniques.

Study participants (44.5 ± 12.3 years [mean ± SD], 54.4% female)		
Detection of morphological findings	Lid transillumination No. (% of eyes)	IRM-OCT No. (% of eyes)
Meiboscore = 0	30 (22.7)	30 (22.7)
Meiboscore = 1	48 (36.4)	48 (36.4)
Meiboscore = 2	30 (22.7)	30 (22.7)
Meiboscore = 3	24 (18.2)	24 (18.2)
Shortening	72 (54.5)	72 (54.5)
Distortion	84 (63.6)	78 (59.1)
Segmentation	54 (40.9)	78 (59.1)
Entanglement	36 (27.3)	36 (27.3)

IRM-OCT = Infrared meibography by optical coherence tomography.

SD = standard deviation.

**Table 3:** Cross-tabulation frequency of the quality of images for interpretation by reflection artifacts.

		Conjunctival reflection (artifact)			
		none	mild	fair	excessive
IRM-OCT images (quality for interpretation)	*Adequate (n = 132)*	78% *(n = 106)*	14.7% *(n = 20)*	4.4% *(n = 6)*	0% *(n = 0)*
	*Unreadable (n = 4)*	0% *(n = 0)*	0% *(n = 0)*	0% *(n = 0)*	2.9% *(n = 4)*

IRM-OCT = Infrared meibography by optical coherence tomography.

### Drop-out (meiboscore)

Of the 132 eyes, no drop-out (meiboscore grade = 0) was present in 30 (22.7%) of the cases, as detected by both techniques. Similarly, meiboscore grade 1, 2, and 3, were detected in 48 (36.4%), 30 (22.7%), 24 (18.2%) of the cases by transillumination biomicroscopy or by IRM-OCT. When comparing drop-out results of the two meibographic techniques, complete agreement was found in 120 (90.9%) of 132 eyes (weighted k = 0.92; *p*<0.001). No discrepancy in classification was observed (McNemar test with Bonferroni correction, *p* = 1.0; Table 4).

**Table 4:** Agreement in meibomian gland detection of morphological changes between meibographic techniques.

	Percentage Agreement No. (% of eyes)	Agreement (Kappa)^a^ 95%CI	Bias in symmetry (*p*-value)^b^
Drop-out (meiboscore)	120 (90.9)	0.92^c^ (0.84–0.99)	1.0^d^
Shortening	120 (90.9)	0.81 (0.57–0.93)	1.0
Distortion	114 (86.3)	0.71 (0.54–0.84)	0.23
Segmentation	108 (81.8)	0.64 (0.48–0.79)	<0.001
Entanglement	120 (90.9)	0.77 (0.61–0.92)	1.0
Microscopicfeatures*	116 (87.8)	0.76 (0.55–0.87)	0.01
All morphological features**	117 (88.6)	0.77 (0.6–0.89)	0.02

^a^ Cohen’s kappa; *p* < 0.001 for all comparisons shown.

^b^ McNemar test (binomial distribution used).

^c^ Cohen’s kappa, weighted; *p* < 0.001.

^d^ Bonferroni correction for *post-hoc* analysis.

* = detection of at least one of the following morphological findings: shortening / distortion / segmentation / entanglement.

** = detection of dropout or/and microscopic features.

### Microscopic features

Of the 132 eyes, shortening, distortion, segmentation, and entanglement, by transillumination biomicroscopy were present in 72 (54.5%), 84 (63.6%), 54 (40.9%), 36 (27.3%) of the cases, respectively. By comparison, shortening, distortion, segmentation, and entanglement, were detected by IRM-OCT in 72 (54.5%), 78 (59.1%), 78 (59.1%), 36 (27.3%) eyes, respectively. Individually, the clinical presence of each microscopic feature demonstrated substantial to almost perfect agreement between results of transillumination biomicroscopy and IRM-OCT (k = 0.81; k = 0.71; k = 0.64; k = 0.77; *p*<0.001, for shortening, distortion, segmentation, and entanglement, respectively). Overall agreement for microscopic features was substantial (k = 0.76; *p*<0.001). However, the majority of disagreement occurred for cases with segmentation, where agreement was present in only 108 (81.82%) of 132 eyes, and where IRM-OCT tended to diagnose more ‘aggressively’ its presence (McNemar test, *p*<0.001; Table 4).

### All morphological features

Overall agreement for *all* morphological features was substantial (k = 0.77; p<0.001, please see Table 4).

### Association analysis

A statistically significant relationship was found between presence of drop-out and shortening, distortion, segmentation, or entanglement (Pearson χ square tests, p<0.001, please see Table 5). Interestingly, segmentation and distortion pattern negatively correlated with the degree of drop-out, whereas shortening and entanglement pattern demonstrated only a weak correlation (Spearman’s ρ was -0.691, -0.491, -0.359, -0.385, respectively, please see Table 5).

**Table 5:** Association and correlation analysis between atrophic processes and microscopic morphological features.

	Drop-out (atrophy) detection	
Shortening	χ = 52.140^a^	ρ = -0.359*
Distortion	χ = 42.361^b^	ρ = -0.491*
Segmentation	χ = 54.688^c^	ρ = -0.691*
Entanglement	χ = 20.075^d^	ρ = -0.385*

χ = Pearson Chi-Square; *p* < 0.001 for all associations shown.

ρ = Spearman Correlation;

^a, b, c, d^ Zero cells (0.0%) have expected count less than 5. The minimum expected count is 10.91, 8.79, 8.79, 6.55, respectively.

* Based on normal approximation.

## Discussion

Traditional methods available for directly studying the architecture of MGs are meibography and biopsy [18]. Clearly, biopsy may reveal microscopic features of glandular structure, but it is an invasive *ex vivo* analysis and patients are frequently reluctant to consent to such procedure. In contrast, a number of contact and non-contact meibographic systems may permit a non-invasive *in vivo* study through direct observation of gross and microscopic features of the silhouette of MGs. Nevertheless, although MGD is one of the most common abnormalities in ophthalmic practice as well as the major cause of evaporative dry eye, or meibography represents the most important diagnostic approach for MGs observation, morphological evaluation of MGs is performed only in a limited number of clinics [16,17].

In the present study, we introduce a simple, novel technique to obtain a noncontact infrared meibography by a commercially available spectral-domain OCT system, which is, to date, one of the most widely used tool in diagnostic imaging in ophthalmology. Using our procedure, the architecture of glandular rows is easily visible in both upper and lower eyelid within 2–3 minutes, without all disadvantages of traditional methods. In particular, patients discomfort induced by the direct application of the transilluminating light probe (contact meibography), or the need for an *expensive*/*dedicated* noncontact meibographic system, for an *indirect digitization* of MGs images, or the topical anesthesia before the invasive alignment of the device lens directly against the everted eyelid (e.g., as in case of meibography obtained by confocal microscopy), are all limits addressed by our method.

Unlike OCT tomograms of MGs previously reported [28–29], our IRM-OCT approach utilizes a standard, commercially available spectral-domain OCT, which is faster than a time-domain (i.e. Visante OCT), and it doesn’t require an *external software* to elaborate images (but only the commercially available, *built-in software* to elaborate OCT images), thereby leading an easier, less time-consuming examination.

The authors believe that the method proposed herein is a useful, outstanding, simple approach to allow meibography to be truly a widespread, routine examination in clinical and research use. In fact, although spectral-domain OCT working at a wavelength of 840 nm (840-nm OCT) was primarily developed to evaluate posterior segment [30], its infrared signal is able to *noninvasively* detect MGs for their peculiar peak of absorbance, related to the tubulo-acinar wall and/or to their lipid content, which appear different from the fibrous tarsus.

With regard to the agreement results, the nearly equal numbers of men and women, inclusion of normal subjects and patients with both microscopic morphological features and MG drop-out, as well as the relatively balanced distribution of glandular atrophic levels supports the external validity of our findings. Although lid transillumination represents a standard technique of contact meibography, its use may still currently provide a non-expensive, well-recognized approach for a number of countries in the world. On the other hand, our technique doesn’t involve any additional cost in diagnostic departments where there is a similar OCT system. Overall, we found substantial agreement between standard meibography and our IRM-OCT method for the detection of all morphological features. Individually, each parameter showed substantial to almost perfect agreement in results between the two techniques, except for segmentation, where bias in symmetry was found to be statistically significant. The failure of standard meibography to strongly correlate with segmentation detected by IRM-OCT in this study suggests that our technique may be more sensitive not only in revealing to such alteration in patients with MGD, but also in detecting a thinning/interruption of glandular wall along MG rows, or a non-homogeneous distribution of lipids in the glandular lumen. We believe that a similar finding may be important for an early diagnosis of subtle morphological alterations of glandular structure during obvious and non-obvious MGD, as well as for a more accurate follow-up.

To further investigate the morphological abnormalities in the MGs, we introduced for the first time two new *well-defined* parameters, segmentation and entanglement. To our surprise, eyes with presence of MG drop-out (grade 1, 2, 3) were significantly associated with entanglement of MG structure, which indicates that atrophic processes may alter the regular architecture of the MGs at any stage of evolution. In contrast, the segmentation pattern was mainly observed in patients with lower drop-out grades, suggesting that the destructive process of glandular rows implies their early fragmentation, which is easily detectable by 840-nm infrared OCT. From a theoretical point of view, segmentation and entanglement are two promising new parameters for evaluating MGD. In this sense, IRM-OCT could effectively demonstrate early abnormalities in glandular morphology before extensive and/or obvious atrophy in their area.

In general, since MGD may be associated with increased risk of ocular complications, i.e. contact lens intolerance and/or discontinuation of contact wear, ocular allergies and ocular surface diseases (e.g., pterygium), discomfort after visual display terminal (VDT) usage, intolerance to glaucoma medications, unsatisfactory refractive surgery outcomes, post-cataract endophthalmitis, etc. the authors firmly believe that IRM-OCT could truly help ophthalmologists in the assessment for prevention and management of all these conditions [31–37].

There are some limitations to the present study. First, although our technique permits direct observation of MGs on OCT, without exporting and processing OCT images with an additional/external software, it implies *manual* adjustments of images according to the reference values described above. Future studies should introduce and evaluate a totally automatic software, which can be easily integrated on OCT systems. Second, further investigations are necessary to clarify the association of our findings with aging and gender, and the diagnostic validity of segmentation and entanglement. Third, we did not perform a comparison analysis between proximal, mid and distal parts of the glandular structure for agreement.

In summary, our novel technique may represent a useful, simple and patient-friendly approach for obtaining information on the MG structure with one of the most widely used device in ophthalmic imaging. Therefore, IRM-OCT has the potential to popularize the field of meibography, facilitating a widespread clinical and research use. Although both meibographic techniques have their advantages (i.e., standard and IRM-OCT), in general there was close agreement between these methods, particularly for MG dropout, which supports the reliability of our novel OCT approach. Specifically, compared with lid transillumination, IRM-OCT seemed more sensitive in detecting microscopic structural changes, e.g. segmentation of glandular rows.

For all these reasons, the procedure described herein may represent an easily accessible method for MG observation and enrich the diagnostic armamentarium of ophthalmologists for studying some very common ocular surface diseases (e.g., MGD, lipid layer deficiency and/or evaporative dry eye).

## Supporting Information

S1 Table Minimum data set. A table with summaries of demographic data of patients.
(XLSX)

S2 Table Descriptive statistics of patients. (DOC)
