# Supplementary material for: A Simple Novel Technique of Infrared Meibography by Means of Spectral-Domain Optical Coherence Tomography: A Cross-Sectional Clinical Study
Source: PLoS One. 2016 Oct 31;11(10):e0165558. doi: 10.1371/journal.pone.0165558 (PMC5087862; doi:10.1371/journal.pone.0165558)
Supplement: S2 Table — (DOC) [file pone.0165558.s002.doc]

| **Descriptive Statistics** | | | | | | | | | | | | | |
| --- | --- | --- | --- | --- | --- | --- | --- | --- | --- | --- | --- | --- | --- |
|  | N | Range | Minimum | Maximum | Sum | Mean | | Std. Deviation | Variance | Skewness | | Kurtosis | |
|  | Statistic | Statistic | Statistic | Statistic | Statistic | Statistic | Std. Error | Statistic | Statistic | Statistic | Std. Error | Statistic | Std. Error |
| Gender_Healthy | 75 | 1,00 | 1,00 | 2,00 | 118,00 | 1,5733 | ,05750 | ,49792 | ,248 | -,303 | ,277 | -1,961 | ,548 |
| Age_Healthy | 75 | 45,00 | 22,00 | 67,00 | 3310,00 | 44,1333 | 1,44413 | 12,50657 | 156,414 | ,390 | ,277 | -1,210 | ,548 |
| Gender_MGD | 61 | 1,00 | 1,00 | 2,00 | 92,00 | 1,5082 | ,06454 | ,50408 | ,254 | -,034 | ,306 | -2,068 | ,604 |
| Age_MGD | 61 | 34,00 | 31,00 | 65,00 | 2755,00 | 45,1639 | 1,55521 | 12,14658 | 147,539 | ,390 | ,306 | -1,430 | ,604 |
| Gender_Overall.Population | 136 | 1,00 | 1,00 | 2,00 | 210,00 | 1,5441 | ,04287 | ,49989 | ,250 | -,179 | ,208 | -1,998 | ,413 |
| Age_Overall.population | 136 | 45,00 | 22,00 | 67,00 | 6065,00 | 44,5956 | 1,05571 | 12,31162 | 151,576 | ,381 | ,208 | -1,295 | ,413 |
| Valid N (listwise) | 61 |  |  |  |  |  |  |  |  |  |  |  |  |
